# Efficiency Enhancement of Electro-Adsorption Desalination Using Iron Oxide Nanoparticle-Incorporated Activated Carbon Nanocomposite

**DOI:** 10.3390/mi12101148

**Published:** 2021-09-24

**Authors:** Ahmed S. Yasin, Ahmed Yousef Mohamed, Donghyun Kim, Sungmin Yoon, Howon Ra, Kyubock Lee

**Affiliations:** 1 Graduate School of Energy Science and Technology, Chungnam National University, Daejeon 34134, Korea; yasin@cnu.ac.kr (A.S.Y.); donghyun4310@o.cnu.ac.kr (D.K.); ysmable@kier.re.kr (S.Y.); 2 IPIT & Department of Physics, Jeonbuk National University, Jeonju 54896, Korea; yousef@jbnu.ac.kr; 3 Korea Institute of Energy Research (KIER), 152 Gajeong-ro, Yuseong-gu, Daejeon 34129, Korea

**Keywords:** AC/Fe_2_O_3_, hydrothermal, electrode, capacitive deionization

## Abstract

Capacitive deionization (CDI) technology is currently considered a potential candidate for brackish water desalination. In this study, we designed iron oxide nanoparticle-incorporated activated carbon (AC/Fe_2_O_3_) via a facile and cost-effective hydrothermal process. The as-synthesized material was characterized using several techniques and tested as electrodes in CDI applications. We found that the distinctive properties of the AC/Fe_2_O_3_ electrode, i.e., high wettability, high surface area, unique structural morphology, and high conductivity, resulted in promising CDI performance. The electrosorptive capacity of the AC/Fe_2_O_3_ nanocomposite reached 6.76 mg g^−1^ in the CDI process, with a high specific capacitance of 1157.5 F g^−1^ at 10 mV s^−1^ in a 1 M NaCl electrolyte. This study confirms the potential use of AC/Fe_2_O_3_ nanocomposites as viable electrode materials in CDI and other electrochemical applications.

## 1. Introduction

With the growth of the population, increasing environmental pollution, and rapid economic development, the demand for affordable clean water is growing rapidly [1]. Owing to the high cost, nonrenewable nature, and environmental hazards resulting from traditional purification methods, it is necessary to search for alternative water purification technologies that are cost-effective and eco-friendly [2]. Consequently, the development of attractive desalination techniques is considered an important challenge for researchers worldwide [3,4]. Over the years, many desalination methodologies have been developed, including electrodialysis, distillation, and reverse osmosis, which are the most commonly utilized and prevalent technologies [5,6]. However, these approaches have several issues, such as high maintenance costs and secondary chemical wastes [7,8]. Considering that brackish water is more prevalent in the world than freshwater, it is attractive to use the large resources of brackish water for human consumption, industry, residential use, and agriculture.

Capacitive deionization (CDI), an innovative and robust water purification methodology in comparison with other deionization techniques, has gained great attention because of its advantages of energy efficiency, rapid regeneration, environmental friendliness, and low cost [9,10,11]. When a certain potential (usually ≤2 V) is applied to the two charged electrodes, the salt ions can be rapidly adsorbed electrostatically on the electrode with two opposite polarities, thus forming an electrical double layer (EDL) and therefore obtaining clean water [12]. The ion removal performance in the CDI process is closely associated with the properties of the electrode materials [13,14]. For decades, many porous carbon materials with high porosity and good conductivity have been widely studied as CDI electrode materials, including carbon nanotubes, carbon aerogels, mesoporous carbon, activated carbon, and reduced graphene [15,16,17].

Activated carbon (AC) possesses unique advantages, including its low price and a high capacity for environmental contaminants [18,19]. However, its higher electrical transfer resistance and lower conductivity limit its development in CDI [20]. AC is usually modified with conductive additives and binders to fabricate CDI electrodes. The first CDI electrodes were fabricated using porous AC, a polymeric binder, and a conductive material [21,22]. Hou et al. claimed that using 10% poly(vinylidenefluoride) (PVDF) as a binder could achieve better performance in CDI experiments [23]. Introducing hydrophilic functional groups via chemical modification methods can also enhance the performance of AC electrodes [24]. To achieve synergistic effects, the combination of different metal oxides with carbon can significantly alter many physicochemical properties of carbon, such as wettability, zeta potential, and surface area, which might greatly improve the CDI performance [25]. Iron oxide, for example, is chemically stable, environmentally benign, and possesses high pseudocapacitance in redox reactions. Thus, iron oxide in the form of carbon composites, especially with AC, could potentially be a good choice for many applications such as energy storage and other related technologies [26,27,28]. Zafra et al. [29] have prepared carbon aerogels doped with manganese or iron oxides via the resorcinol-formaldehyde method. The capacitance values as high as 99 F/g and 91 F/g were respectively obtained for CAGDFeAct and CAGDMnAct via cyclic voltammetry. The large capacitance obtained for the iron containing activated aerogel was affirmed via deionization experiments. An electrosorption capacity of 0.133 mmol/g was obtained for CAGDFeAct in a 0.025 M NaCl solution at 1.5 V during the charge period. Belaustegui et al. [30] have prepared a three-dimensional reduced graphene oxide decorated with iron oxide nanoparticles (3D rGO-Fe_2_O_3_) material that possesses a suitable porous structure via a one-step hydrothermal treatment. The fabricated material exhibits a large specific capacitance (345.13 F g^−1^ at 5 mV s^−1^) and high adsorption capacity (945.1 mg g^−1^ for 11,700 mg L^−1^ NaCl solutions) compared with the other carbon materials presented in the literature for CDI electrodes. The incorporation of iron oxide nanoparticles was beneficial for enhancing the specific capacitance as well as the specific surface area of 3D rGO, which achieved a much higher NaCl uptake during the CDI process. These achieved results clearly indicated effectiveness of these novel approaches to further improve the performance of iron oxide nanoparticles-based carbon material for CDI application.

Motivated by the above promising reports, herein this work, iron oxide nanoparticles were elaborately incorporated onto activated carbon through an easy and safe method with increased mass production and interesting morphology. The synthesized nanocomposite was examined as efficient material for desalinating salty water through the CDI process. The synergistic effect between iron oxide nanoparticles and activated carbon was beneficial for enhancing the desalination performance of the fabricated nanocomposite. A superior wettability, specific capacitance, and favored electrosorptive capacity were attained. AC/Fe_2_O_3_ also displayed a lowered charge transfer resistance to illustrate the facile electron transfer step at its surface during the studied CDI experiment. Accordingly, this present work could provide helpful guidance on developing efficient CDI electrode material for promising performance.

## 2. Experiments

### 2.1. Materials and Methods

Activated carbon (CEP-21K, PCT Co., Gumi-si, Korea) and iron (III) nitrate nonahydrate (Sigma-Aldrich, St. Louis, MO, USA) were used without any treatment. A Nafion^®^ solution (Sigma-Aldrich, St. Louis, MO, USA) was used in the electrochemical studies. A glassy carbon electrode (GCE; with 0.071 cm^2^ area) (CH Instruments, Inc., Austin, TX, USA) was also used.

### 2.2. Synthesis of Activated Carbon/Fe_2_O_3_ Nanocomposite

The typical synthetic procedure for AC/Fe_2_O_3_ was as follows: 0.65 g of AC was first dispersed via ultrasonication in 80 mL of deionized water for 1 h to obtain solution A. Subsequently, Fe(NO_3_)_3_·9H_2_O (0.08 g) was totally dissolved in 10 mL of deionized water via vigorous stirring for 15 min to obtain solution B. Solutions A and B were then loaded into a Teflon-lined autoclave that was then filled up to 80% of its total volume with deionized water. Thereafter, the autoclave was sealed at a controlled temperature of 120 °C and maintained for 24 h. Finally, the obtained solution was cooled naturally until it reached room temperature. Afterwards, the product was washed four times at room temperature with distilled water in order to remove any impurities; after that, it was dried at 60 °C for 12 h to obtain the final product.

### 2.3. Characterization

The morphology of the AC/Fe_2_O_3_ composite was investigated using field emission scanning electron microscopy (FE-SEM) (Hitachi S-7400, Tokyo, Japan). The shape of the AC/Fe_2_O_3_ was analyzed using transmission electron microscopy (TEM) (JEM-2100 F HR, JEOL, Tokyo, Japan) with energy dispersive X-ray (EDX) spectroscopy. The structure of AC/Fe_2_O_3_ was examined using X-ray diffraction (XRD) (Rigaku, Tokyo, Japan). The elemental composition and electronic structure of AC/Fe_2_O_3_ were investigated using X-ray photoelectron spectroscopy (XPS) (AXIS-NOVA, Kratos Analytical Ltd., Manchester, UK). The Brunauer–Emmett–Teller (BET) surface areas for the fabricated materials were investigated by measuring their nitrogen adsorption (BELSORP Mini II, MicrotracBEL, Osaka, Japan). 

### 2.4. Electrochemical Properties of the Synthesized Electrode

Cyclic voltammetry experimental measurement was carried out using different NaCl solutions (1 M), and the sweep potential range was adjusted from −0.4 to 0.6 V (versus Ag/AgCl) in an electrochemical cell with a three-compartment cell at room temperature containing the prepared nanocomposite electrode, a platinum wire, and Ag/AgCl electrode as working, counter-electrode, and reference electrode, respectively. The surface of carbon support was pre-treated by polishing with alumina slurries and cleaning with ethanol double-distilled water mixture. A portion of 2 mg of this nanocomposite powder was homogenized into 420 μL isopropanol with adding 20 μL Nafion and sonicating this suspension at 40 °C for 30 min. The glassy carbon electrode surface was then left to dry in an air oven at 80 °C for 10 min. A VersaStat 4 Potentiostat device ZIVE SP1 (WonATech Co. Ltd. Seoul, Korea) and the VersaStudio software program were utilized for controlling the system. The specific capacitance could be calculated by integrating the full CV cycle to determine the average value according to the following relationship:

$$C_{s}=\;\frac{\int i\;dV}{2v∆Vm}$$

where *C_s_* is the specific capacitance (F g^−1^), *i* is the response current (A), *V* is the potential (V), *υ* is the potential scan rate (V s^−1^), and *m* is the mass of the electro-active materials in the electrode (g). A frequency response analyzer (FRA) connected to a VersaStat 4 Potentiostat device was used for measuring the electrochemical impedance spectroscopy (EIS) in the similar cell in the abovementioned setup. The amplitude of the alternating voltage was 5 mV around the equilibrium potential (0 V) and had a frequency range of 0.01–10 KHz.

### 2.5. Electrosorptive Capacity Measurement

The CDI electrodes were fabricated by mixing (80 wt.%) of the modified activated carbon and polytetrafluoroethylene (20 wt.%), followed by ultrasonication for 1 h in order to test the total electrosorptive capacity of the CDI system through the proposed carbon material. The slurry mixture was coated onto a carbon electrode after it dried at 100 °C overnight. The CDI electrode (5 cm × 5 cm) was obtained with a 40 mg/cm^2^ geometric surface area. The electrode was used as the working electrode. A NaCl aqueous solution, with an initial concentration of 50 mg/L and total volume of 30 mL, was supplied to the cell. Three potential values of 1.0, 1.2, and 1.4 V were adjusted to judge the relative performance of the whole CDI cell.

The salt removal efficiency (η), the electrosorptive capacity (*Sc*) of the electrode, and average salt absorption rate (*ASAR*) can be calculated according to the following equations:

$$\eta =\left(\frac{Co-C}{Co}\right)\times 100\%$$

Sc=(Co−C)V/m

$$ASAR=\frac{Sc}{t}$$

where *Co* and *C* (mg/L) are the initial and final NaCl concentrations, respectively, *V* (L) is the total volume of the NaCl aqueous solutions, *m* (g) represents the mass of the active components in the working electrodes, and *t* is time of deionization process.

## 3. Results and Discussion

**Figure 1:**
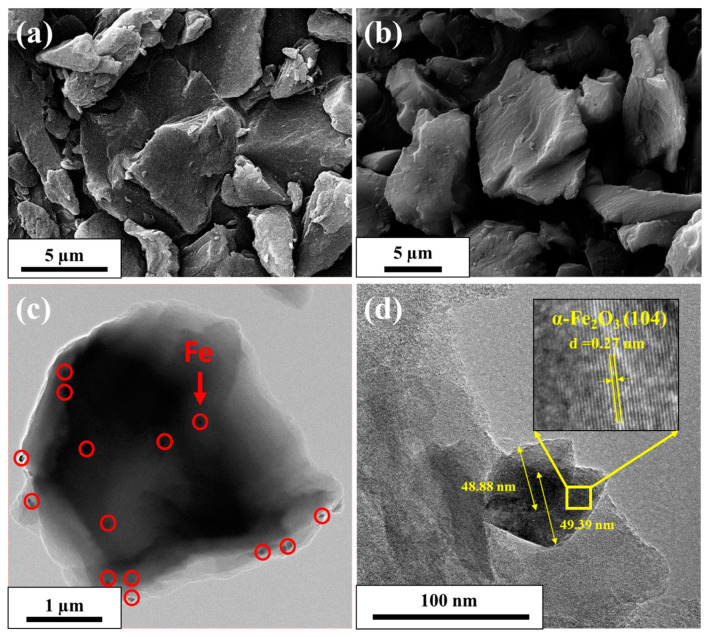
FE-SEM images of (**a**) AC and (**b**) AC/Fe_2_O_3_ nanocomposite. (**c**,**d**) TEM images of AC/Fe_2_O_3_ nanocomposite.

In addition to improving its electrical conductivity and the overall electrochemical properties, modifying AC with Fe_2_O_3_ nanoparticles can play a crucial role in improving the physicochemical characteristics of AC, thus achieving an efficient CDI electrode. SEM and TEM images of pristine AC and the AC/Fe_2_O_3_ nanocomposite are shown in Figure 1. Both AC and AC/Fe_2_O_3_ have micron-sized particles with rock-like shapes. The TEM images of the AC/Fe_2_O_3_ nanocomposite (Figure 1c,d and Figure S1a–d) show the homogeneous incorporation of Fe_2_O_3_ nanoparticles throughout the AC sheet, with an average particle size of ~55 nm. The inset image in Figure 1d describes the HRTEM of the produced nanoparticles, which show the regular arrangement of the atoms to form an atomic plane. The interplanar spacing is found around 0.27 nm, which is in fully consistent with the 104 plane of the main peak of Fe_2_O_3_ (Table S1).

**Figure 2:**
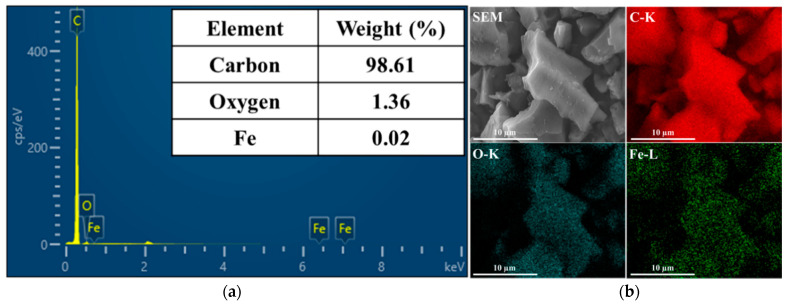
(**a**) TEM-EDX analysis and (**b**) elemental mapping of the AC/Fe_2_O_3_ nanocomposite.

The TEM-EDX analysis results are shown in Figure 2a. The analysis indicates that the AC/Fe_2_O_3_ nanocomposite contained carbon as the main constituent (98.61%), oxygen (1.36%), and iron (0.02%). Although these percentages do not reveal the real ratios of the fabricated nanocomposite as in EDX, they confirm the presence of Fe_2_O_3_. TEM mapping confirmed the homogeneous distribution of C, O, and Fe in the composite particles (Figure 2b).

**Figure 3:**
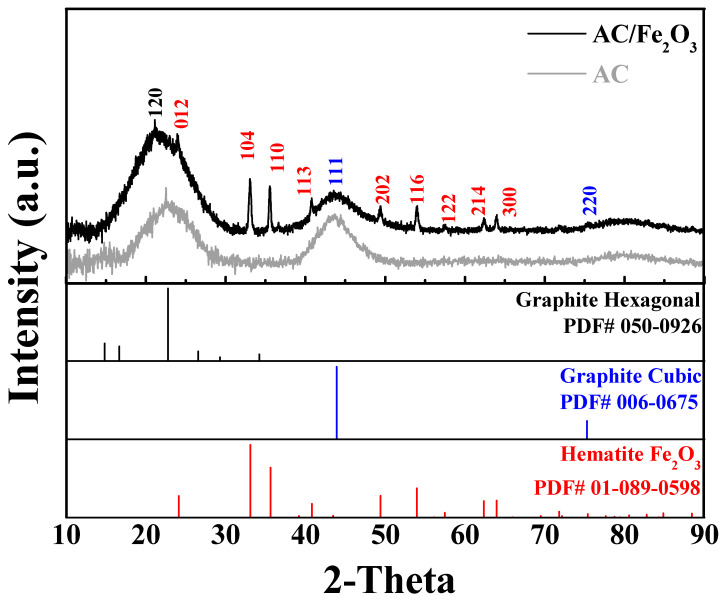
XRD patterns of AC and AC/Fe_2_O_3_ nanocomposite.

The XRD patterns of the AC and AC/Fe_2_O_3_ composite are presented in Figure 3. The XRD pattern for AC used as a reference material clearly shows the changes in the peaks when Fe_2_O_3_ was incorporated on its surface. AC exhibits an amorphous structure with two broad peaks at 2θ of approximately 22.7°, and 43.7° corresponding to the (120) planes of the graphite hexagonal structure and the (111) cubic structure, respectively. In the case of AC/Fe_2_O_3_, additional Bragg peaks of Fe_2_O_3_, the hematite phase, are clearly observed.

It is known that the XRD line broadening is affected by the internal strain and crystallite size. The Scherrer method was therefore used to determine the crystal size. The average crystal size of the Fe_2_O_3_ nanoparticles was approximately 49 nm. The lattice parameters of Fe_2_O_3_ were in good agreement with the hematite reference card, which proves the efficient incorporation of hematite Fe_2_O_3_ on the AC surface (Table 1, Figure 1c,d). The specific surface area and pore-size distributions of the AC/Fe_2_O_3_ nanocomposite were investigated using N_2_ sorption isotherms and the Barrett–Joyner–Halenda (BJH) technique, as illustrated in Figure S2. The AC/Fe_2_O_3_ nanocomposite possessed a high surface area of 2042.9 m^2^ g^−1^ and a pore size of approximately 1.88 nm. These results show that the AC/Fe_2_O_3_ nanocomposite is superior to the iron oxide-based carbon composites reported in the literature [31,32]. The obtained high surface area is expected to shorten the ion diffusion pathways, thus improving the specific capacitance [33].

**Figure 4:**
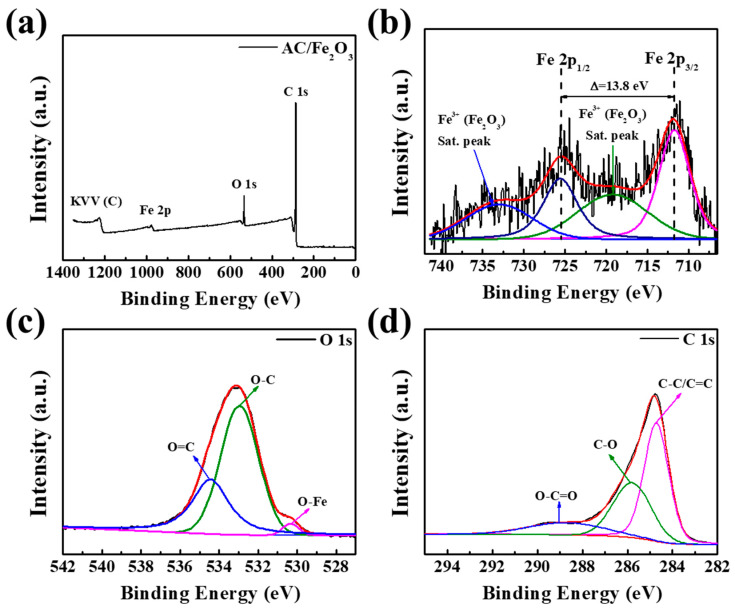
(**a**) XPS survey spectra and deconvoluted spectra of (**b**) Fe 2p, (**c**) O 1s, and (**d**) C 1s.

XPS analysis was performed to further characterize the surface structure of the modified AC/Fe_2_O_3_. Figure 4a shows a typical XPS survey spectrum for AC/Fe_2_O_3_. The XPS profile shows peaks of Fe 2p, O 1s, and C 1s; no other elemental peaks were detected, which reveals that the AC/Fe_2_O_3_ mainly included three elements: Fe, O, and C. In the fitted Fe 2p spectrum depicted in Figure 4b, there are two broad peaks at 711.78 and 725.48 eV, assigned to Fe 2p_3/2_ and Fe 2p_1/2_, respectively. The satellite peaks at 719.08 and 733.18 eV, which correspond to the main peaks of Fe 2p_3/2_ and Fe 2p_1/2_, respectively, were assigned to the Fe^3+^ in Fe_2_O_3_, which agrees well with previous reports [34,35]. The peak positions of the three parts divided in the O 1s XPS spectrum (Figure 4c) were 530.38 eV (Fe–O), 532.98 eV (O–C), and 534.48 eV (O=C), indicating that the Fe_2_O_3_ was incorporated efficiently on the AC surface based on the obtained chemical bonds. The C 1s XPS spectrum of AC/Fe_2_O_3_ in Figure 4d exhibits three major peaks with binding energies at 284.78, 285.88, and 289.28 eV, which are consistent with the C–C/C=C, C–O, and O–C=O configurations, respectively [36,37]. Furthermore, the interference of Fe metal was further confirmed by the atomic concentration (atomic weight %) presented in Table 2. In conclusion, the refined XPS spectral analysis results suggest the successful incorporation of Fe_2_O_3_ nanoparticles on the AC surface.

**Figure 5:**
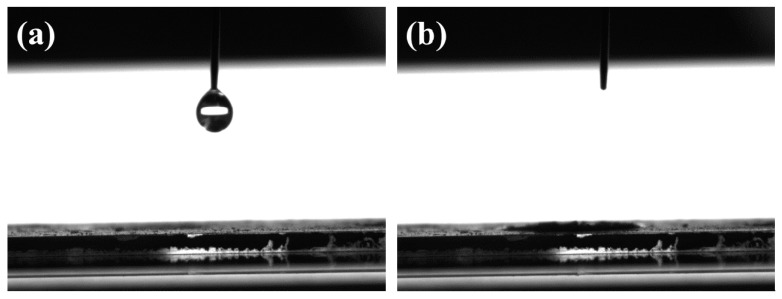
WCA for the AC/Fe_2_O_3_ nanocomposite (**a**) before and (**b**) after release of a water droplet.

The wettability of AC and the AC/Fe_2_O_3_ nanocomposites was evaluated through water contact angle (WCA) experiments. Photographic images of the water drops on the material surfaces are shown in Figure 5 and Figure S3. According to the literature, the wettability of the surface is associated with the hydrophobic/hydrophilic properties of the utilized material and its components [38]. The WCA value of AC was 86.8° (Figure S3). The WCA dramatically decreased when Fe_2_O_3_ species were incorporated into AC, below 3°, as depicted in Figure 5a,b. The enhanced wettability revealed the successful incorporation of Fe_2_O_3_ nanoparticles on the AC particles, as inferred from the abovementioned XPS analysis. It is predicted that the increased surface free energy would enhance ion diffusion within the nanocomposite structure during the desalination process [39,40]. For comparison, Trinh et al. [31] investigated the wettability of Fe_3_O_4_ nanoparticles intercalated in reduced graphene oxide (rGO). The hydrophilicity of this carbon-based material was improved after the modification of the rGO with Fe_3_O_4_ nanoparticles in different ratios (Fe_3_O_4_/rGO): 0:1 (51.7°, bare rGO), 1:8 (49.5°), 1:4 (26.1°), and 1:2 (34.8°). This indicates the successful incorporation of Fe_2_O_3_ on AC by the hydrothermal method in our study.

**Figure 6:**
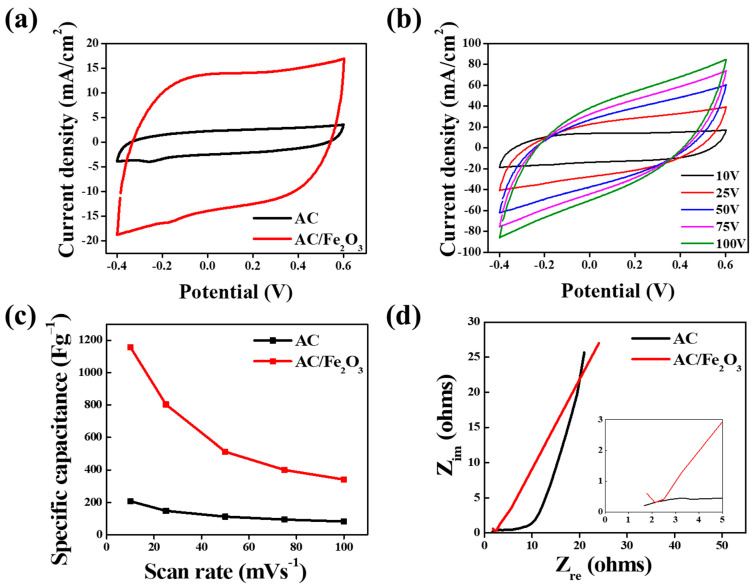
CV curves (**a**) for the proposed electrodes and (**b**) for the AC/Fe_2_O_3_ electrode at various sweep rates. (**c**) Specific capacitance for the electrodes and (**d**) Nyquist plot for the electrodes in a 1 M NaCl aqueous solution.

The CV tests were performed within a potential window range of −0.4 to 0.6 at various scan rates in 1 M NaCl, and the obtained results are depicted in Figure 6. To study the influence of Fe_2_O_3_ nanoparticles on AC, comparative CV curves for AC and the AC/Fe_2_O_3_ nanocomposites were obtained at a sweep rate of 50 mV/s (Figure 6a). The larger area obtained from the CV curves suggested the higher specific capacitance of the electrodes. The AC/Fe_2_O_3_ nanocomposite exhibited a CV curve with a much larger area than AC, suggesting a higher specific capacitance than that of AC. The curves revealed rectangular voltammetric behavior in both AC and the AC/Fe_2_O_3_ nanocomposites, indicating that both electrodes possessed excellent EDL performance owing to coulombic interactions [41]. Figure 6b shows the CV curves for the AC/Fe_2_O_3_ nanocomposite at various sweep rates ranging from 10 to 100 mV/s. The CV curves revealed a common shape of the capacitive characteristics originating from the coulombic reaction. As the applied sweep rate was increased, the CV curves deviated from the rectangular shape accordingly. The specific capacitance was estimated based on the CV curves for both the AC and AC/Fe_2_O_3_ electrodes. The plot of the specific capacitance versus sweep rate is shown in Figure 6c. Much higher specific capacitance values were delivered by the AC/Fe_2_O_3_ electrode (1157.5, 802.74, 512.33, 400, and 341.1 F g^−1^) than by the AC electrode (207, 147, 111, 94, and 82 F g^−1^) at sweep rates of 10, 25, 50, 75, and 100 mV s^−1^, respectively. The Nyquist profiles representing the EIS of AC and AC/Fe_2_O_3_ are shown in Figure 6d. In the high-frequency region, the AC/Fe_2_O_3_ curve exhibits a smaller semicircle than the AC curve, which means that AC/Fe_2_O_3_ possessed a smaller electronic transfer resistance. Additionally, the steeper slope for AC/Fe_2_O_3_ obtained at low frequencies indicates faster diffusion of the salt ions in both electrodes [42]. Considering the longer time spent during the electrosorption–desorption process for AC/Fe_2_O_3_ than for AC, ion diffusion within the AC/Fe_2_O_3_ electrode may not significantly affect the CDI performance.

**Figure 7:**
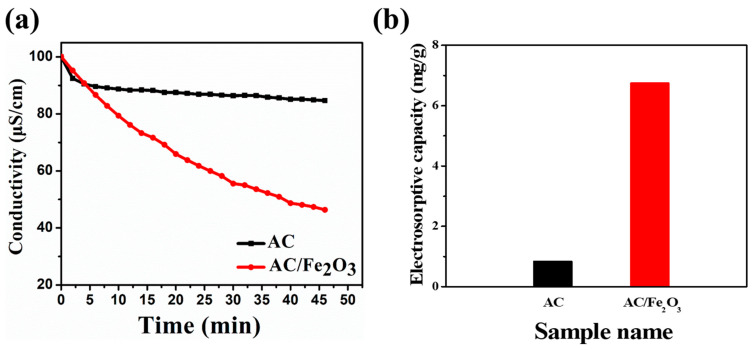
(**a**) CDI profiles, (**b**) electrosorption capacity for the fabricated electrodes.

The desalination performances of AC and AC/Fe_2_O_3_ electrodes at 1.2 V were tested in a NaCl solution (~100 μs cm^−1^). As shown in Figure 7a, once the voltage was applied to the CDI cell, the ions in the salt solution migrated to the oppositely charged electrodes and were absorbed into the pores of the active electrode material, resulting in a dramatic decrease in the conductivity. Clearly, the AC/Fe_2_O_3_ nanocomposite showed a significantly higher ion adsorption capacity than the AC electrode. The CDI cell with the AC/Fe_2_O_3_ electrode achieved a high salt removal efficiency (*η*) of 53.68%. The CDI with the AC electrode exhibited a salt removal efficiency of only 15.29%. The electrosorptive capacities were calculated to be 0.85 mg g^−1^ for the AC electrode and 6.76 mg g^−1^ for the AC/Fe_2_O_3_ electrode (Figure 7b). Moreover, the average salt absorption rate was calculated to be 0.018 mg g^−1^ min^−1^ for the AC electrode and 0.14 mg g^−1^ min^−1^ for the AC/Fe_2_O_3_ electrode. Figure S4 depicts the regeneration profiles for the AC/Fe_2_O_3_ electrode. The introduced AC/Fe_2_O_3_ can be regenerated completely and easily, which ensures a cost-effective and low energy future for the CDI process. Notably, the AC/Fe_2_O_3_ nanocomposite electrode showed a higher electrosorption capacity than previously reported relevant electrodes, as shown in Table 3. This remarkable performance obtained for the AC/Fe_2_O_3_ electrode is ascribed to the enhanced wettability after the incorporation of AC with Fe_2_O_3_ nanoparticles, which might facilitate ion adsorption based on the full contact between the AC/Fe_2_O_3_ electrode and the salt solution. Additionally, its high specific surface area effectively produced more sites for an optimum adsorption of a large number of ions. The outstanding electrical conductivity of the AC/Fe_2_O_3_ nanocomposite is also a crucial parameter for achieving high capacitance.

## 4. Conclusions

An AC/Fe_2_O_3_ nanocomposite-based CDI electrode was developed by incorporating Fe_2_O_3_ onto AC using a facile as well as a cost-effective hydrothermal method, which showed enhanced performance in electro-adsorption desalination. The AC/Fe_2_O_3_ nanocomposite electrode showed a high electrosorptive capacity (6.76 mg g^−1^ at 1.2 V) and an 8-fold enhancement compared with the AC electrode, and it outperformed previously reported AC-based composite electrodes. Based on diverse characterization results, we attribute its superior performance to the good dispersion of Fe_2_O_3_ nanoparticles on the surface of the AC sheet and high wettability and conductivity, which resulted in remarkable electrochemical performance. Cyclic voltammograms showed the higher capacitance of AC/Fe_2_O_3_ at a low sweep rate (1157.5 F g^−1^ at 10 mV s^−1^) than that of AC only. As a result, the electrolyte conductivity of AC/Fe_2_O_3_ rapidly decreased during the CDI desalination process, suggesting a higher adsorption capacity for the composite. This research finding might pave a new pathway to the development of nanocomposite materials not only for desalination but also for various electrochemical applications, such as electrodes for supercapacitors or flexible batteries.

## Figures and Tables

**Table 1:** Lattice parameters of Fe_2_O_3_.

Lattice Parameters	Hematite, Fe_2_O_3_ (Ref.)	AC/Fe_2_O_3_
Crystal system	Rhombohedral	Rhombohedral
Space group	*R*3*c*	*R*3*c*
a	5.038 (Å)	5.041 (Å)
b	5.038 (Å)	5.041 (Å)
c	13.776 (Å)	13.73 (Å)

**Table 2:** The atomic concentration of XPS analysis of AC/Fe_2_O_3_ nanocomposite.

Name	Peak BE	FWHM (eV)	Atomic %
C 1s	284.82	1.84	90.57
Fe 2p	711.45	2.56	0.30
O 1s	533.17	2.87	8.96

**Table 3:** The specific capacitance and electrosorption capacity for different carbon-based electrodes.

Electrode Material	Specific Capacitance (F g^−1^)/Scan Rate (mV s^−1^)	Applied Voltage (V)	Initial Concentration (mg L^−1^)	Electrosorption Capacity (mg g^−1^)	Ref.
MC	251/1	1.2	25	0.68	[43]
CNTs-MC	132.6/10	1.2	40	0.69	[44]
RG-CNTs	175/1	2	~27	1.41	[45]
AC	169/1	1.2	~25	0.25	[43]
RG-AC	181/1	1.2	~500	2.94	[46]
AC-MnO_2_	77.2/10	1.2	~25	0.99	[47]
AC/TiO_2_ NPs	―	1.2	500	2.7	[48]
AC/Fe_2_O_3_	1157.5/10	1.2	~50	6.76	This work

## Supplementary Materials

The following are available online at https://www.mdpi.com/article/10.3390/mi12101148/s1, Figure S1: TEM images of the AC/ Fe_2_O_3_ nanocomposite; Figure S2: N_2_ adsorption-desorption isotherm, and pore size distribution curve of the AC/ Fe_2_O_3_ nanocomposite; Figure S3: Water contact angel with the function of the contact time for the AC; Figure S4: Regeneration profile for the AC/Fe_2_O_3_ electrode; Table S1: XRD analyses of the fabricated AC/Fe_2_O_3_ nanocomposite (Fe_2_O_3_-peaks).
