# Supplementary material for: Efficiency Enhancement of Electro-Adsorption Desalination Using Iron Oxide Nanoparticle-Incorporated Activated Carbon Nanocomposite
Source: Micromachines (Basel). 2021 Sep 24;12(10):1148. doi: 10.3390/mi12101148 (PMC8539726; doi:10.3390/mi12101148)
Supplement: Supplementary file 1 [file micromachines-12-01148-s001.zip › micromachines-1383307-supplementary.pdf]

Efficiency Enhancement of Electro-adsorption Desalination using Iron Oxide Nanoparticles-incorporated Activated Carbon Nanocomposite

Ahmed S. Yasin [a], Ahmed Yousef Mohamed [b], Dong Hyun Kim [a], Sung Min Yoon [c], Ho Won Ra [c]*, and Kyubock Lee [a]*

[a] Graduate School of Energy Science and Technology, Chungnam National University, Daejeon, 34134, Republic of Korea

[b] IPIT & Department of Physics, Jeonbuk National University, Jeonju 54896, Republic of Korea

[c] Korea Institute of Energy Research (KIER), 152 Gajeong-ro, Yuseong-gu, Daejeon, 34129, Republic of Korea

*Corresponding author. Tel: +82 42-821-8610. E-mail: seojun@kier.re.kr (H. W. Ra), kyubock.lee@cnu.ac.kr (K. Lee)

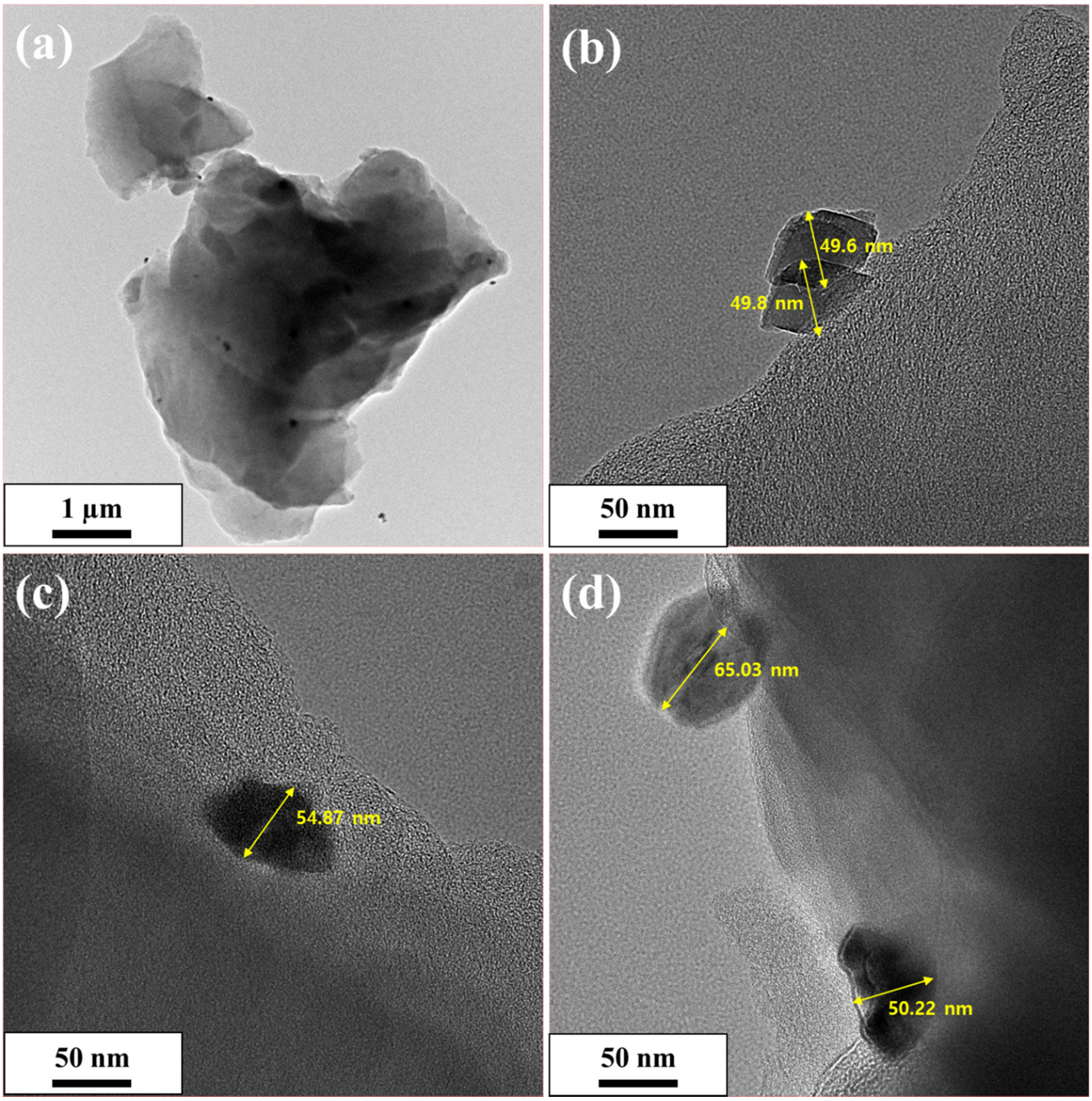

Figure S1. (a-d) TEM images of the AC/ Fe_2O_3 nanocomposite.

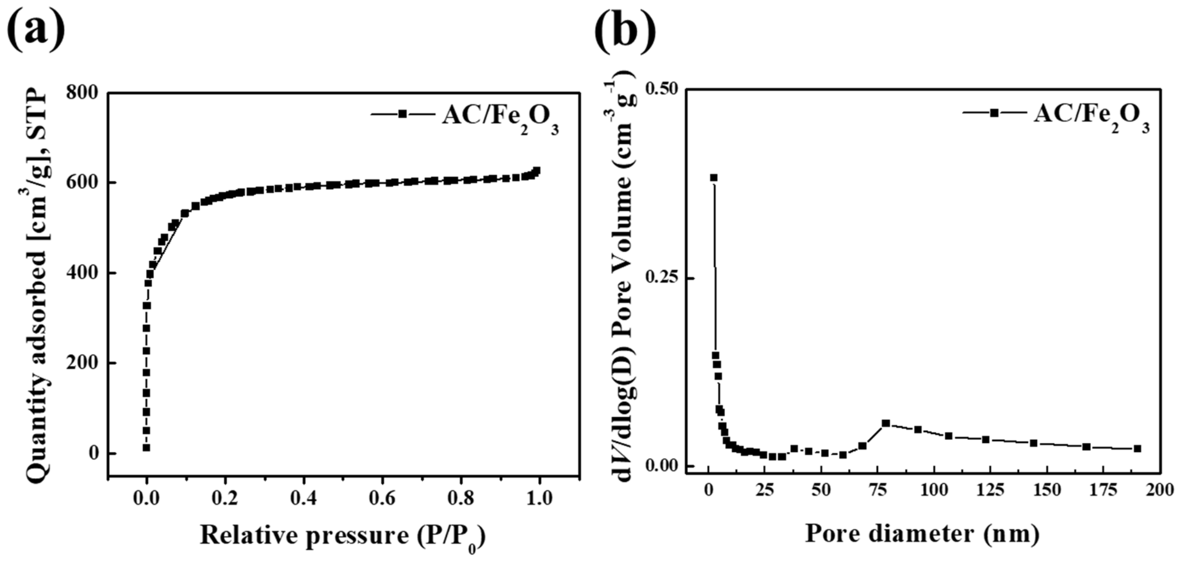

Figure S2. (A) N_2 adsorption-desorption isotherm, and (B) pore size distribution curve of the AC/ Fe_2O_3 nanocomposite.

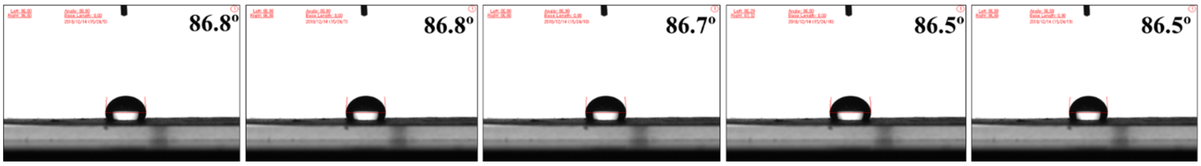

Figure S3. Water contact angel with the function of the contact time for the AC.

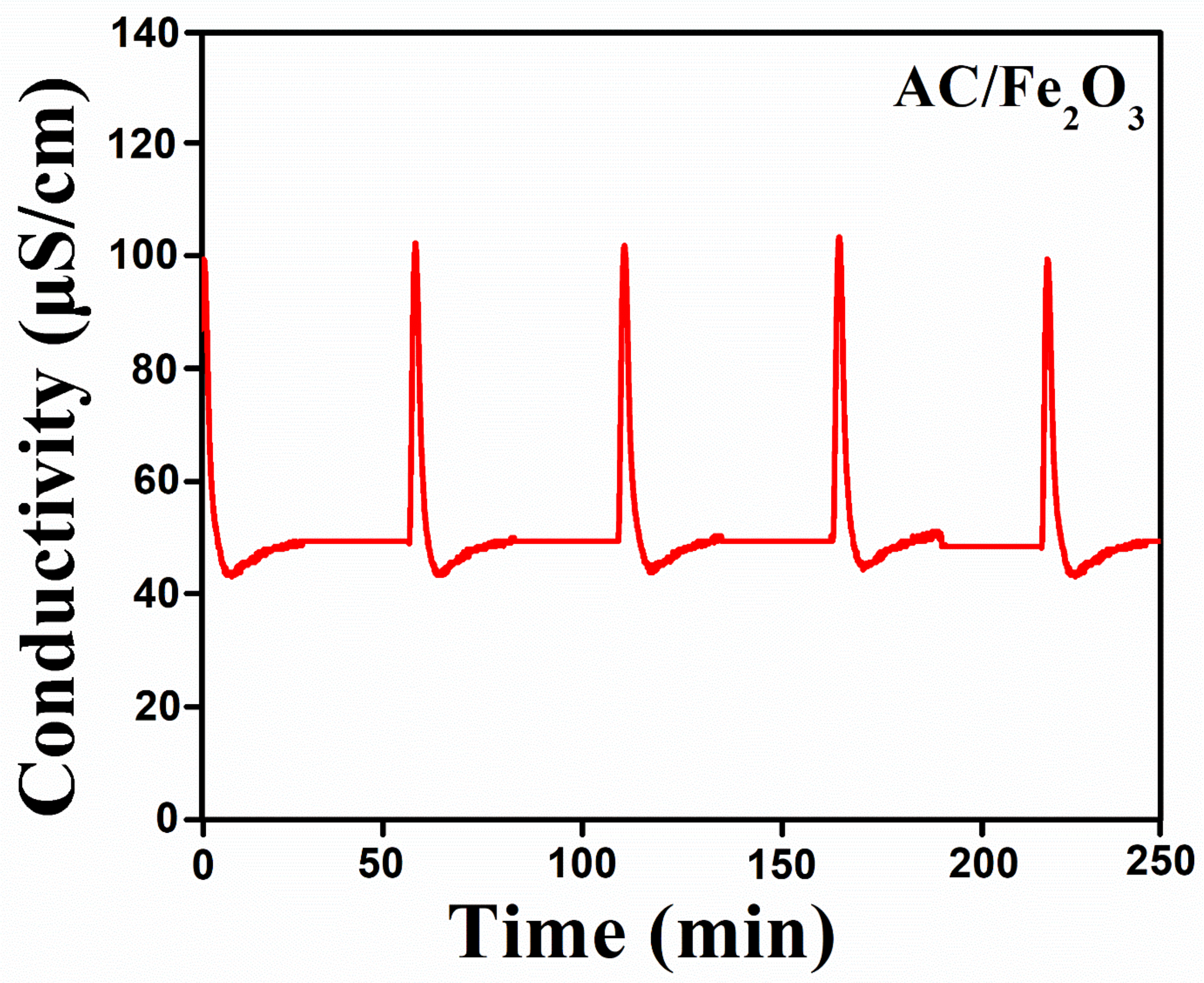

Figure S4. Regeneration profile for the AC/Fe_2O_3 electrode.

Table S1. XRD analyses of the fabricated AC/Fe_2O_3 nanocomposite (Fe_2O_3-peaks).

No.	2θ (degree)	d-spacing (from JCPDS)	Miller Indices (h k l)	Relative Intensity (%)
1	24.13	3.686	0 1 2	29.7
2	33.11	2.703	1 0 4	100
3	35.61	2.519	1 1 0	69
4	39.21	2.296	0 0 6	2.2
5	40.83	2.209	1 1 3	19.1
6	43.48	2.08	2 0 2	2.5
7	49.41	1.843	0 2 4	29.8
8	54	1.697	1 1 6	40.3
9	56.13	1.637	2 1 1	0.2
10	57.49	1.602	1 2 2	6.4
11	62.38	1.487	2 1 4	22.5
12	63.96	1.454	3 0 0	23.5
13	65.97	1.415	1 2 5	0.1
14	69.49	1.352	2 0 8	2
15	71.8	1.314	1 0 10	7.8
16	72.15	1.308	1 1 9	1.8
17	75.41	1.26	2 2 0	4.6
18	77.65	1.229	0 3 6	1.7
19	78.72	1.215	2 2 3	0.9
20	79.44	1.205	1 3 1	0.1
21	80.6	1.191	3 1 2	2.8
22	82.8	1.165	0 2 10	3.9
23	84.29	1.148	0 0 12	0.2

24	84.86	1.142	1 3 4	5.6
25	88.47	1.104	2 2 6	5.2
